# Three case reports of West Nile virus neuroinvasive disease: lessons from real-life clinical practice

**DOI:** 10.1186/s12879-021-06827-9

**Published:** 2021-11-03

**Authors:** Camilla Falcinella, Marina Allegrini, Lidia Gazzola, Giovanni Mulè, Daniele Tomasoni, Ottavia Viganò, Antonella d’Arminio Monforte, Giulia Marchetti, Camilla Tincati

**Affiliations:** Clinic of Infectious Diseases and Tropical Medicine, Department of Health Sciences, ASST Santi Paolo e Carlo, University of Milan, Milan, Italy

**Keywords:** West Nile virus, Arbovirus, Neuroinvasive disease, Cerebrospinal fluid, Case report

## Abstract

**Background:**

Despite being an uncommon cause of meningoencephalitis, West Nile virus (WNV) recently provoked significant outbreaks throughout Europe. West Nile neuroinvasive disease (WNND) is associated with significant morbidity and mortality in older and compromised individuals, while its diagnosis may be demanding for the clinician. Here discussed are three cases of WNND with a focus on the diagnostic challenges they presented due to atypical clinical presentation and laboratory findings.

**Case presentation:**

Between July and September 2020 three patients presented to our attention with signs and symptoms compatible with meningoencephalitis. Among routine procedures, they underwent lumbar puncture and imaging. In the absence of microbiological isolates, biological samples were sent for serology and NAATs for WNV. Following diagnosis, the patients gradually recovered and were discharged either home or to rehabilitation facilities.

**Conclusions:**

The laboratory findings here discussed, in particular CSF parameters, are only partially consistent with those described in the literature, which highlights the need for further research. While serology and NAATs on blood and urine appear the most reliable techniques in the diagnostic work-up of WNND, utility of NAATs on CSF specimens is limited by the kinetics of WNV viremia in biological fluids. This report underlines that WNND should always be included in the differential diagnosis of meningoencephalitis during WNV transmission period.

## Background

West Nile virus (WNV) has established an endemic autochthonous transmission in Europe, particularly within the Mediterranean area. Often described as a re-emerging disease, WNV numbers and geographic extension have increased, possibly due to climate change and its ecologic consequences [1, 2]. In 2018, an overall 7.2-fold rise in WNV infections was reported in Europe, notably in Bulgaria, France and Italy; in 2020 316 cases were recorded in Europe, of which 68 in Italy [3, 4].

Infection occurs predominantly through mosquito bites, from July to September in temperate regions. Symptoms develop in 20% of patients with either West Nile fever or neuroinvasive disease (WNND). WNND is more likely to occur in older and compromised subjects, especially in the encephalitic form. Clinically, it is similar to other aseptic meningoencephalitides, with modest CSF lymphocytosis, elevated proteins and normal glycorrhachia, and is associated with a considerable morbidity/mortality, particularly in the older subjects with comorbidities. Notably, a consistent proportion of patients complain of persistent long-term neurologic dysfunction, mainly within the cognitive domain [5, 6].

Here presented are three WNND cases managed at our department between July and September 2020. Their clinical presentation and laboratory findings significantly differ from traditional descriptions, which highlights knowledge gaps in the clinical and diagnostic work-up of neuroinvasive WNV infection.

## Case presentation

### Patient#1

A 68-year-old man presented to our institution at the end of July with a one-week history of fever with no apparent organ involvement except for a diffuse skin rash, interpreted as allergic reaction to amoxicillin/clavulanate. His wife reported a few episodes of confusion in correspondence to fever. His past medical history was significant for anxiety, pituitary adenoma and prostatectomy for a carcinoma.

On examination the patient was alert, oriented, febrile and markedly asthenic, with fine crackles at the pulmonary bases, mild abdominal tenderness and a petechial rash involving the four extremities, more marked on the lower limbs, and no signs of meningeal irritation. Evaluation revealed neutrophilic leukocytosis, lymphopenia (970/mm^3^), mildly elevated CRP (19 mg/L) and pulmonary fibrotic alterations on CT scan. A BGA showed hypoxemia and respiratory alkalosis. Empirical antibiotic therapy with ceftriaxone was started.

Over the following days he developed progressive psychomotor slowing, persisting fever and worsening abdominal tenderness. Laboratory testing showed neutrophilic leukocytosis (12,980/mm^3^) with a rise in CRP (101.8 mg/L). Acute abdominal causes were excluded, while general infectious workup returned negative. Our attention was thus drawn to the patient’s worsening cognitive status, described as perfectly conserved just until hospital admission. Neurological examination revealed diplopia, positive Babinski sign bilaterally and resistance to head passive mobilization in the absence of frank meningeal signs. He couldn’t sit in the bed or walk autonomously and complained of headache and cervicalgia. Brain CT and MRI scan were negative, bilateral slow-wave activity on central-anterior leads was shown by EEG; a lumbar puncture was performed (Table 1). Empirical therapy with meropenem and acyclovir was prescribed.

**Table 1:** CSF findings in our three patients compared to those described in the literature

	Tyler et al. 2006 [7]	Patient#1	Patient#2	Patient#3
Macroscopic characteristics	N/A	Limpid	Limpid	Limpid, slight xanthochromia
RBC count [cells/mm^3^]	200 (M), 545 (E)	< 5	N/A	1000
WBC count [cells/mm^3^]	226 (M), 227 (E)	195	66	760
WBC subpopulation	45% (M) and 37% (E) predominantly PMNs	Predominantly mononuclear	Almost exclusively mononuclear	PMNs
Proteins [mg/dL]	76 (M), 101 (E)	158	59	430
Glucose [mg/dL]	65 (M), 71 (E)	50	61	46
CSF/serum glucose ratio	N/A	0.43	0.55	0.42

*M* meningitis, *E* encephalitis, *PMNs* polymorphonuclear leukocytes, *N/A* not available, glycorrhachia: normal range 65–100 mg/dL, CSF/serum glucose ratio: normal range > 0.6

On day 4, the patient’s cognitive status and temperature started improving; microbiological tests and exfoliative cytology on CSF turned out negative. On day 8, WNV infection was diagnosed by positive serology (both IgG and IgM) as well as serum and urine PCR (2108 and 129,686 copies/mL, respectively), while frozen CSF tested negative (Table 2).

**Table 2:** Diagnostic work-up in our three patients; here shown are the results of the single laboratory test and the time between symptom onset and sample collection

	Serum IgM	Serum IgG	PCR—blood	PCR—urine	PCR—CSF
Patient#1	+ / 12 days	+ / 12 days	+ / 12 days	+ / 12 days	− / 8 days
Patient#2	+ / 10 days	+ / 10 days	+ / 10 days	− / 10 days	− / 5 days
Patient#3	+ / 8 days	− / 8 days	+ / 8 days	+ / 8 days	N/A

*N/A* not available

Symptoms progressively resolved and his neurologic exam normalized, with the exception of mild cognitive dysfunction and frontal headache; he rapidly regained the ability to walk autonomously and was discharged on day 13.

### Patient#2

In August, a 66-year-old man was evaluated for impaired consciousness and a 6-day history of headache, cervical pain, nausea and low-grade fever. His past medical history was unremarkable. He reported a stay on a lakeside locality about one month before. Since his retirement he used to do some outdoor gardening.

Routine work-up showed normal WBC and differential count, negative CRP and no other alterations. Brain CT scan was negative, while his neurological examination showed slight weakness of the right upper limb in the absence of frank meningeal signs. CSF findings are shown in Table 1. Empirical therapy with ampicillin, ceftriaxone and acyclovir was started. The following day the patient was afebrile, alert and oriented, with no neurologic abnormalities. CSF microscopic examination resulted negative, as did PCR for all the major bacterial and viral pathogens. On EEG no alterations were found except for a minimal excess of slow-wave activity on temporal leads bilaterally. Brain MRI and routine infectious work-up were negative.

On day 7 of hospitalization diagnosis of WNND was made by positive blood PCR (552 copies/mL). WNV serology was positive for IgM and IgG, while PCR on frozen CSF and urine was negative (Table 2).

Two days later he was discharged, asymptomatic except for mild psychomotor slowing.

### Patient#3

In September, an 85-year-old woman was admitted for a 4-day history of fever and dysuria with a previous diagnosis of lower limb thrombophlebitis, treated at home with azithromycin and low-molecular-weight heparin. Her daughter described the development of a rash after the start of antibiotic therapy. A single, self-limiting episode of disorientation was reported. The patient suffered from hypertension, hypothyroidism and asthma.

**Fig. 1:**
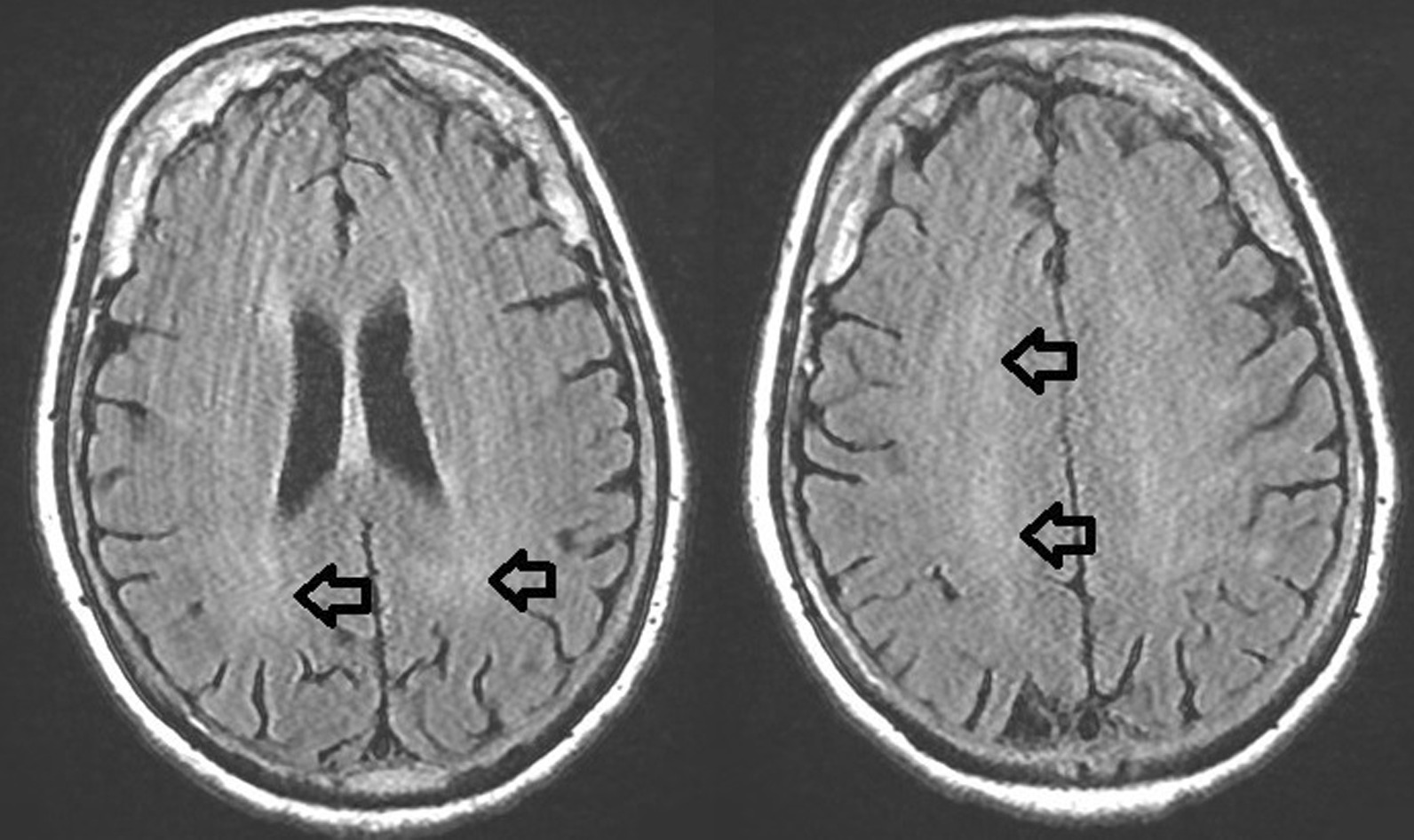
Patient#3’s brain MRI performed at 16 days from symptoms onset, showing lobar white matter hyperintensities (black arrows) on T2-FLAIR sequences. Motion artifacts are present

On a first evaluation she was afebrile, eupneic but hypoxic in ambient air, with no evident abnormalities on physical examination except for slight bilateral lower limb edema. Shortly after, the patient became agitated, hyperpyretic and oliguric; abdominal tenderness was detected. Initial workup revealed a normal WBC count with slight neutrophilia, CRP elevation and increased D-dimer (943 ng/mL). Pulmonary embolism and pneumonia were excluded by a contrast-enhanced CT scan. In the emergency department her conditions deteriorated with persistence of high-grade fever and worsening mental status. Neurologic exam revealed nuchal rigidity with cervical pain upon flexion, diffuse tremors on the four limbs, poorly comprehensible speech and impaired execution of simple tasks, in the absence of focal neurologic signs. Increased CRP (68.3 mg/L) and neutrophilic leukocytosis were shown. A lumbar puncture was performed with subsequent CSF examination (Table 1). Treatment with vancomycin, meropenem and antiepileptic medications was prescribed. Meanwhile, all microbiological studies on blood, urine and CSF resulted negative. On reappraisal a focal vision defect was noticed and further investigations were performed: on EEG bilateral slow-wave alterations were recorded on central-anterior leads, while brain MRI revealed irregular, non-specific lobar white matter hyperintensities on T2-weighted sequences (Fig. 1).

After an initial worsening of the patient’s conditions with development of a comatose state and persistence of involuntary movements, her level of consciousness started improving, albeit residual impairment of cognitive and motor functions.

On the 12th day of hospitalization, PCR for WNV tested positive on blood and urine (4171 and 8,989,833 copies/mL, respectively), while serology was positive only for IgM (Table 2). No CSF samples were available for examination. On day 19, she was transferred to a rehabilitation facility.

## Discussion and conclusions

We describe three WNND cases in unrelated patients all living in the southern section of Milan metropolitan area, two of which in close proximity to the Naviglio Grande fluvial canal.

Clinical, laboratory and instrumental findings are summarized in Table 3.

**Table 3:** Main clinical, laboratory and instrumental findings regarding the three cases here described

	Symptoms	Neurological exam	CSF finding	MRI findings	EEG findings	Management	Outcome
Patient#1 68 yo Male Caucasian	Fever, confusion, skin rash	Diplopia, bilateral positive Babinski sign, resistance to head passive mobilization	Mononuclear pleocytosis, hyper-protidorrachia, hypo-glycorrachia	No abnormalities reported	Bilateral slow-wave activity on central-anterior leads	Supportive care; suspension of empiric ABT on diagnosis confirmation	Mild cognitive impairment followed by complete recovery; no relapses reported
Patient#2 66 yo Male Caucasian	Headache, cervical pain, nausea, low-grade fever	Slight right upper limb weakness	Mononuclear pleocytosis, slightly increased proteins, hypo-glycorrachia	No abnormalities reported	Minimal bilateral slow-wave activity on temporal leads	Supportive care; suspension of empiric ABT on diagnosis confirmation	Mild cognitive impairment followed by complete recovery; no relapses reported
Patient#3 85 yo Female Caucasian	Fever, dysuria, skin rash, disorientation	Nuchal rigidity, diffuse tremors, slurred speech, impaired task execution, focal vision defect	Increased RBC count, polymorphonuclear pleocytosis, hyper-protidorrachia, hypo-glycorrachia	Lobar white matter hyperintensities on T2-weighted sequences	bilateral slow-wave alterations were recorded on central-anterior leads	Supportive care; suspension of empiric ABT on diagnosis confirmation; antiepileptic therapy	Significant motor/cognitive impairment followed by gradual recovery; no relapses reported

*ABT* antibiotic therapy; RBC red blood cell

From a clinical standpoint, the picture of neuroinvasive disease may be ill-defined. Patient#1 and #3 presented no early and overt signs and symptoms of meningoencephalitis, thus challenging the suspicion of WNND. Further, CSF findings presented inter-subject variability and were only partially coherent with data on 250 patients with WNND (Table 1). Strikingly, while normal glycorrhachia is traditionally reported in WNND, we observed reduced glucose level in all patients, both as absolute value and CSF/serum glucose ratio. Albeit reflecting disease severity and/or the small number of subjects, our result disputes the prognostic value of CSF findings, which is currently debated [7]. Neutrophilic pleocytosis was detected in patient#3 who underwent lumbar puncture precociously, a finding consistent with early polymorphonuclear infiltrate followed by lymphocytic predominance [5, 6].

Head CT scans were normal in all cases. MRI showed signal alterations only in patient#3 (Fig. 1), reflecting data on MRI positivity after several weeks after illness onset, and poorer prognosis in patients with MRI anomalies [5, 8]. While thalamic, basal ganglia or internal capsule involvement is most commonly described, T2/FLAIR abnormalities within white matter structures, as in our third case report, have been reported by other case series [9]. Significant residual cognitive impairment was present in all subjects upon discharge, highlighting that the long-term burden of disease may affect the quality of life and everyday functioning.

Two subjects displayed cutaneous manifestations, interpreted as hypersensitivity reactions. WNV-related rash is more frequently observed in younger subjects and thought to be protective against WNND [10]. Our findings suggest that skin rash in older subjects with WNND may be more frequent than reported and stimulate a reassessment of its prognostic significance in this context.

The etiologic diagnosis of WNND may also pose significant difficulties. Indeed, WNV-RNA was not retrieved in CSF samples (Table 2), despite positive serology and blood PCR in all cases. Although inefficient WNV-RNA amplification cannot be excluded, our findings confirm the limited sensitivity of virus isolation/NAATs given possible pathogen clearance by the time of illness onset [5]. Further, in accordance with reports showing detectable WNV-RNA at a higher load and for a longer time in the urine than in the blood, two patients tested positive for urine WNV PCR (Table 2), thus supporting the use of urine NAAT techniques in the diagnostic work-up of WNV [11–13]).

In conclusion, WNV infection is a too often neglected but endemic disease in Europe, with periodic epidemic bursts that might become more and more frequent in the years to come. Through the present case reports, we mean to shed light on some little-known aspects of the infection, particularly on the discrepancies between real-life scenarios and traditional case descriptions found in the literature, which highlight the need for further research. Above all, this is a reminder for the clinician that a high level of suspicion should be maintained for the whole duration of WNV transmission period, irrespective of signs and symptoms at presentation.

## Data Availability

The data that support the findings of the current study are available from the corresponding author upon reasonable request.

## Abbreviations

BGA: Blood gas analysis
CRP: C-reactive protein
CSF: Cerebrospinal fluid
CT: Computed tomography
EEG: Electroencephalogram
MRI: Magnetic resonance imaging
NAAT: Nucleic acid amplification test
PCR: Polymerase chain reaction
RNA: Ribonucleic acid
WNND: West Nile virus neuroinvasive disease
WNV: West Nile virus
